# Assessment of European health professionals’ educational needs in basic principles of geriatric medicine: a focus group qualitative analysis from the PROGRAMMING COST Action 21122

**DOI:** 10.1007/s41999-026-01430-0

**Published:** 2026-03-11

**Authors:** Rachael Frost, Ana Viegas, Georgios-Konstantinos Tsamasiotis, Mitilda Gugu, Efterpi Mougakou, Sumru Savas, Robert Kupis, Karolina Piotrowicz, Susana Ganhão-Arranhado, Ana Farinha, Anna Marie Herghelegiu, Ovidiu Lucian Bajenaru, Catalina Raluca Nuta, João Fonseca, Anna Rudzińska, Vesna Popov, Pavlinka Milosavljevikj, Vasiliki Sakellari, Nilufer Demiral Yilmaz, Helena Lesz-Przybył, Ana-Gabriela Prada, Maria Tampaki, Laura M. Pérez, Yolanda Barrado-Martín, Christina Avgerinou, Maja Ortner Hadziabdic, Anna Christakou, Eleni Moumtzi, Stefan Arsov, Santiago Cotobal Rodeles, Evrydiki Kravvariti, Marina Kotsani, Tamar Yellon

**Affiliations:** 1 School of Public and Allied Health, Liverpool John Moores University, Tithebarn Building 79 Tithebarn Street, Liverpool, L2 2ER UK; 2 Unidade Local de Saúde Do Algarve, E.P.E. Rua Leão Penedo, 8000-386 Faro, Portugal; 3 Physiotherapy Department, University of West Attica, Athens, Greece; 4 Department of Preclinical Subjects, Faculty of Medical Technical Sciences, University of Elbasan “Aleksandër Xhuvani”, Elbasan, Albania; 5 First Department of Internal Medicine, Korgialenio-Benakio Hellenic Red Cross General Hospital, Athens, Greece; 6 Geriatrics Section, Department of Internal Medicine, School of Medicine, Ege University, Izmir, Türkiye; 7 Department of Medical Education, Centre for Innovative Medical Education, Jagiellonian University Medical College, Kraków, Poland; 8 Department of Internal Medicine and Gerontology, Faculty of Medicine, Jagiellonian University Medical College, Kraków, Poland; 9 Faculty of Health Sciences and Nursing, Centre for Interdisciplinary Research in Health (CIIS), Universidade Católica Portuguesa, 1649-023 Lisbon, Portugal; 10 Atlântica, Instituto Universitário, Fábrica da Pólvora de Barcarena, 2730-036 Barcarena, Portugal; 11 CINTESIS, Centre for Health Technology and Services Research, 4200-450 Porto, Portugal; 12 Instituto de Ciências Biomédicas Abel Salazar da, Universidade Do Porto, Portugal: Unidade Local de Saúde da Arrábida, Porto, Portugal; 13 “Carol Davila” University of Medicine and Pharmacy, Bucharest, Romania; 14 “Ana Aslan” National Institute of Gerontology and Geriatrics, Bucharest, Romania; 15 Egas Moniz Health Alliance, Universidade de Aveiro, 3810-193 Aveiro, Portugal; 16 Unidade Local de Saúde da Região de Aveiro, Av. Artur Ravara 35, 3810-164 Aveiro, Portugal; 17 Specialized Hospital for Geriatric and Palliative Care “13t, November”, Skopje, North Macedonia; 18 Department of Palliative Care, PHU Specialized Hospital for Geriatric and Palliative Medicine “13t, November”, Skopje, North Macedonia; 19 Department of Medical Education, School of Medicine, Ege University, Izmir, Türkiye; 20 Department of Geriatrics, Faculty of Health Sciences, Medical University of Silesia, Katowice, Poland; 21 Faculty of Medicine, “Carol Davila” University of Medicine and Pharmacy, Bucharest, Romania; 22 Department of Geriatrics and Gerontology, Hospital of Chronic Diseases “Saint Luke”, Bucharest, Romania; 23 Internal Medicine Department, Thoracic Diseases General Hospital Sotiria, Athens, Greece; 24 Research Group On Aging, Frailty and Transitions, Vall d’Hebrón Institut de Recerca (VHIR) and Parc Sanitari Pere Virgili, Barcelona, Spain; 25 Research Department of Primary Care and Population Health, University College London, London, UK; 26 Research Department of Primary Care and Population Health, University College London, London, UK; 27 Faculty of Pharmacy and Biochemistry, University of Zagreb, Zagreb, Croatia; 28 Department of Physiotherapy, University of Peloponnese, Sparta, Greece; 29 Physical Medicine and Rehabilitation Department, 414 Military Hospital of Special Diseases, Athens, Greece; 30 Faculty of Medical Sciences, Goce Delcev University, Stip, North Macedonia; 31 Geriatric Medicine Department, University Hospital of Salamanca, Salamanca, Spain; 32 First Department of Propaedeutic Internal Medicine, School of Medicine, National and Kapodistrian University of Athens, Athens, Greece; 33 LNA Santé, Cagnes-Sur-Mer, France; 34 Hellenic Society for the Study and Research of Aging, Athens, Greece; 35 Jerusalem College of Technology, Jerusalem, Israel; 36 School of Nursing in the Faculty of Medicine, Henrietta Szold Hadassah Hebrew University, Jerusalem, Israel

**Keywords:** Education, Qualitative, Geriatric medicine

## Abstract

**Purpose:**

In many countries, geriatrics is still emerging as a speciality, with limited training options for healthcare professionals (HCPs). This international qualitative study aimed to explore the geriatric educational needs of HCPs, focussing on countries where geriatric medicine (GM) is emerging or underdeveloped.

**Methods:**

Fourteen focus groups (*n* = 125 participants, 82% female, mean age 41.6 years) were carried out representing ten European countries and a range of HCPs and settings. Focus group discussions were recorded, transcribed verbatim, and inductively coded in the original language. Codes were aggregated into a shared English codebook applied to all transcripts. Descriptive and subsequently analytical themes were then developed.

**Results:**

Three themes were identified. (1) Current experiences of geriatric care: participants described caring for older adults as medically and socially complex, requiring strong communication skills and interprofessional collaboration. (2) Structural and contextual challenges: limited staffing, fragmented care pathways, and GM’s emerging status hindered effective care delivery. (3) Uncertainties and unmet training needs: key uncertainties included distinguishing ageing from disease, applying assessment tools, recognising red flags, and safe medication management. Participants emphasised the need for practical training during undergraduate studies, standardised interprofessional courses for non-specialists, and structural support (e.g. accessible programmes, protected time for training).

**Conclusion:**

Currently, geriatrics training is limited and there are multiple contextual challenges, especially in countries with emerging GM. There is significant unmet need for HCP basic training in GM. Our study contributes to an empirical basis for developing relevant and feasible GM training for HCPs. Structural support is needed for these initiatives.

**Supplementary Information:**

The online version contains supplementary material available at 10.1007/s41999-026-01430-0.

## Key summary points

**Aim:**

To qualitatively explore the geriatric educational needs of healthcare professionals across Europe in the context of current care practices, with a particular focus on countries where geriatric medicine remains underdeveloped.

**Findings:**

The medical and social complexity of geriatric medicine requires developing skills in communication and interprofessional collaboration, as well as focussing on the priority clinical topics of medication management, treatment adaptation, comprehensive assessment, ageing physiology and recognition of red flags. Future training should include practical skills, case-based learning, and didactic content. Strong national and institutional support is needed to ensure consistent, high-quality, and accessible training and care.

**Message:**

There are clear educational gaps for European healthcare professionals working with older people.

**Supplementary Information:**

The online version contains supplementary material available at 10.1007/s41999-026-01430-0.

## Introduction

As chronic disease and disability prevalence rises, demand grows for complex older adult care, requiring substantial adaptations in policy, resources, infrastructure, and workforce planning [1]. Quality geriatric care demands a holistic, person-centred, and integrated approach, as exemplified by the World Health Organization’s (WHO) Integrated care for Older People (ICOPE) model and geriatric medicine (GM) principles [2]. GM specialises in the care for older people, especially those with complex health issues, through a holistic approach and multiprofessional team collaboration [3].

The development of GM as a speciality, discipline, and care model varies substantially across Europe [4–6]. At the midpoint of the current United Nations Decade of Healthy Ageing [7], and in the context of persistent workforce shortages, pragmatic solutions and scalable strategies are needed to ensure high-quality, equitable access to care for older adults across Europe and to reduce regional disparities. In addition to developing GM as a speciality, training all professionals who care for older adults is essential [8]. Education is a core pillar to optimise workforce capacity and performance in the WHO Health Workforce Framework 2030 [9].

Effectively and sustainably upskilling healthcare professionals (HCPs) to provide quality care for older people requires a concerted effort to integrate comprehensive geriatric content throughout the educational continuum [10–15]. However, there is a lack of consensus regarding the optimal delivery of geriatric content [16] and these efforts need to be adapted to HCPs’ educational needs and real-life practice context. Kern’s Six-Step Curriculum Development Framework [17] proposes initial steps of problem identification and targeted needs assessment, of which empirical data collection is a key part.

PROmoting GeRiAtric Medicine in countries where it is still eMergING (PROGRAMMING) COST Action 21122 [18] is a European networking initiative aiming to identify the appropriate content of education and training activities on fundamental principles of GM destined for HCPs practising across various clinical settings, focussing on countries where GM is still under development [19]. As part of its initial phase, PROGRAMMING conducted a mixed-methods needs assessment, combining a large-scale web-based survey [20] with qualitative approaches to capture the experiential and contextual dimensions of geriatric education needs. The present study aims to qualitatively understand the geriatric educational needs of HCPs across Europe, with a focus on real-world clinical practice and health system contexts. By providing an empirically grounded understanding of perceived gaps and priorities, this work seeks to inform the development of relevant, adaptable training frameworks to support the delivery of high-quality geriatric care across diverse European settings where GM is emerging.

## Methods

Focus groups (FGs) were selected to generate rich, in-depth discussions, allowing for the exploration of shared experiences, diverse perspectives, and the nuanced social dynamics within different healthcare contexts relevant to geriatric care and curricula [17, 21]. Fourteen FGs were conducted April 2023–January 2025, primarily in European countries where geriatric medicine (GM) is at an earlier stage of development or implementation. These included single-country FGs in Greece, North Macedonia, Poland, Portugal, and Romania, as well as multi-country focus groups focussed on specific professional types from Albania, Croatia, Greece, Italy, North Macedonia, Poland, Romania, Spain, and Türkiye. This facilitated both cross-country and cross-professional comparisons of GM education needs.

Experts from the UK provided methodological training and oversight for the FGs, but did not contribute participant data, as GM is already well established there. Participants were recruited through professional networks affiliated with the PROGRAMMING Action, using purposive sampling to ensure diversity in professional background and experience.

The topic guide was initially developed by the Greek team (for the first FG) and subsequently refined with minor contextual adaptations for each FG, while maintaining a consistent core structure across all countries and covering the broad topics outlined in Table 1. Each FG was facilitated by two researchers (see Supplementary File 1) with complementary professional backgrounds and expertise, who attended standardised qualitative methods training delivered by RF, YBM, and CA as part of the PROGRAMMING Action. Most participants did not know each other or the facilitators professionally.

**Table 1:** Topics covered in topic guide

• What geriatric care means and who should provide it
• Clinical experiences working with older people, reflecting on real-life patients
• Uncertainties experienced in recent clinical care, and what could help manage this
• Sufficiency of current geriatric clinical knowledge, skills, and training
• Skills and knowledge that require further development
• Impact of (in)sufficient knowledge/training on practice
• How geriatrics education could be incorporated into previous training and current career/setting

Note: there were some minor differences in topic guide questions adapted for different countries and contexts

Ten FGs were carried out in person at Action meetings in the native language of the country; the four multi-country FGs were carried out online in English using Zoom with a high security level. All participants provided informed consent prior to participation, and ethical approval for the study was obtained in accordance with national guidelines and institutional requirements in each participating country, including:

- Greece: Research Ethics Committee, University of West Attica, Athens, Greece: 37149 (05/04/2023).
- Israel: Jerusalem College of technology ethical committee approval: 014_23 (covered approval for all multi-country focus groups).
- North Macedonia: Ethics Committee of the PHI Specialized Hospital for Geriatric and Palliative Care 13th November, Skopje: 03-339/1 (06/02/2024).
- Poland: Ethics Committee of Jagiellonian University Medical College: 118.0043 (01/07/2025).
- Portugal: in light of prior ethical approvals obtained in other participating countries, separate ethical approval was not deemed necessary.
- Romania: ethics was not needed.

All FGs were audio recorded and transcribed verbatim by team members. Reflexivity was encouraged throughout the research process. The analysis approach used principles of codebook thematic analysis [22], to take a partly inductive approach whilst ensuring consistency across coders and language. Each team of facilitators inductively coded transcripts in the original language, then constructed a codebook in English. RF aggregated all supplied codebooks into an overarching thematic framework intended to descriptively summarise the findings under key themes relevant to the research aim. The consolidated codebook was then reapplied by teams to the original transcripts using MS Excel and English summaries of each code were provided in a shared document. RF subsequently integrated data from different countries to generate overall themes and subthemes relating to the research question. An audit trail of coding decisions was maintained in shared documents to ensure dependability. Draft themes were reviewed by all co-authors, ensuring investigator triangulation, and a revised thematic structure was discussed and agreed, incorporating interpretive analysis of cross-cultural patterns. No new themes emerged with the addition of the final two FGs, suggesting saturation had been achieved. Supplementary File 2 summarises the descriptive codebook and subsequent analytical themes.

## Results

Fourteen FGs across ten countries were carried out with 5–13 participants per group (total *N* = 125 participants), including a range of professionals across a variety of acute and community settings (Table 2, Supplementary File 1). Across all focus groups and countries, geriatric care was seen as a substantial part of most healthcare specialties. FG participants were predominantly female (102/125, 82%), with a mean age of 41.6 years. Although they varied in their level of expertise in care of older people, they all described it as being a large part of their work.

**Table 2:** Summary of focus group composition

Single country				
Greece	2	18	Primary care, hospital, rehabilitation	Physicians, nurses, allied health professionals
North Macedonia	2	24	Specialised hospital, long-term care	Physicians, nurses, caregivers, social workers
Poland	2	21	Hospital, outpatient, primary care	Geriatricians, internists, nurses, paramedics
Portugal	2	23	Primary care, hospital	Physicians, nurses, allied health professionals
Romania	2	16	Ambulatory care, long-term care	Geriatricians, GPs, nurses, allied health professionals
**Multi-country**				
Mixed (online)	4	23	Diverse (hospital, primary care, academia)	GPs, internists, geriatricians, nurses, pharmacists
Total	14	125		

Multi-country				
Mixed (online)	4	23	Diverse (hospital, primary care, academia)	GPs, internists, geriatricians, nurses, pharmacists
Total	14	125		

Three themes and nine subthemes were identified (see Table 3).

**Table 3:** Themes and subthemes

1. Current experiences of providing geriatric care:
1a. Balancing clinical complexity with person-centred goals
1b. The importance of communication with patients and their caregivers
2. Structural and contextual challenges:
2a. Resources and accessibility issues
2b. Fragmented healthcare systems
2c. The emerging nature of geriatric care
3. Uncertainties and unmet training needs:
3a. Lack of an evidence base and context-adapted guidelines
3b. Gaps in existing training
3c. Clinical practice uncertainties
3d. Recommendations for a framework to promote geriatric education

### 1a. Current experiences of providing geriatric care: balancing clinical complexity with person-centred goals

Older adults were perceived to have complex medical needs, further complicated by a wide range of non-medical needs (e.g. social, financial, transport, functional), which should also be addressed for effective care. Multidisciplinary working was considered vital to facilitate this.

“[name], a 96-year-old lady, is hospitalized, a social issue has no one who wants her… A lady who has a very great feeling of loneliness and has great support from nurses and therapists.” (Portuguese FG1 Primary care professionals).

The most important geriatric care goals were seen as improving quality of life and optimising health and well-being, as well as preventing any decline from hospitalisation or overtreatment. Supporting healthy ageing and taking a preventative approach where possible were also considered important, focussing on maintaining or increasing autonomy, independence, and a sense of purpose.

“I would like to find a way to help them [older patients] change their daily life for the better, for as long as it lasts.” (Greek FG2, Rehabilitation centre and hospital professionals).

In addition, participants described the emotional labour associated with geriatric care as an integral aspect of managing clinical complexity. HCPs in Greek, Romanian, and Portuguese FGs acknowledged an emotional investment associated with watching decline, patient suffering, management difficulties, and end of life care, particularly where relationships had been built.

“We don’t want to see this ending … we think about the good things and the rainbow, telling ourselves that it’s OK to walk and go home.” (Portuguese FG2, Hospital professionals).

### 1b. Current experiences of providing geriatric care: the importance of communication with patients and their caregivers

Effective, respectful communication with both older people and their caregivers was consistently described as foundational to high-quality geriatric care. Participants however reported variation in how respectful and empathetic communications could be, with instances of inappropriate interactions. Several barriers to shared decision-making were identified, including older people’s poor health literacy, cognitive problems, lack of family support or being non-verbal, which required patience and additional time.

“From my experience as a family doctor, I noticed that communication with older patients requires more time and a diversification of methods. Many of them have multiple health conditions and may also lack adequate family support.” (Romanian FG1, Ambulatory care professionals).

Single-country FG participants felt families played a crucial role in supporting older people, but caregiver involvement varied, posing challenges like inconsistent attendance, excessive control, decision-making strain, resistance to advice, and pressure for inappropriate care.

“I make a lot of home visits and I know that I will turn my back and everything I said will be called into question by the caregiver.” (Portuguese FG1, Primary care professionals).

Caregivers therefore needed to be supported and communicated well with, but there was a perceived lack of information for caregivers across most countries. HCPs found it difficult to provide appropriate advice and support to caregivers when they perceived gaps in their own geriatric knowledge, highlighting communication as both a clinical and educational challenge. These communication challenges directly influenced the ability to deliver person-centred, goal-oriented geriatric care.

### 2a. Structural and contextual challenges: resources and accessibility issues

Participants consistently described staffing problems, and therefore access to certain professional services and specialties (particularly allied HCPs such as physiotherapists or psychologists), as major barriers to delivering timely and appropriate geriatric care. This was compounded by the need for longer consultation times or home-based care for older people. There was a noticeable gap in specialised geriatric care and lack of staff with experience, knowledge, or expertise in working with older people.

“Initially, we only recruited people who had this Geriatrics course… but we reached a time when there were no people in the market.” (Portuguese FG1, Primary care professionals).

This led to safety concerns and reduced confidence in multidisciplinary team (MDT) colleagues, as well as increased pressures on hospital-based care due to a lack of primary, community, and home-based care services.

“Where do we channel all the people? To the hospital.” (Portuguese FG2, Hospital professionals)

Geographic barriers and regional inequalities (e.g. islands, rural areas) also limited the availability and quality of care, which could be further compounded by financial difficulties and a lack of adequate patient transport.

“And here, work, in such an area [rural] we very often hit this wall, this transport wall.” (Polish FG1, outpatient, primary care and hospital professionals).

Teleconsultations were discussed as a potential strategy to improve access in some contexts (Polish FG1); however, participants emphasised that their usefulness was limited for older people with hearing impairment or cognitive difficulties.

### 2b. Structural and contextual challenges: fragmented healthcare systems

Participants consistently described fragmentation across healthcare settings as a major obstacle to coordinated and effective geriatric care. Although multidisciplinary working was considered critical to address the multi-faceted needs of older people, this was substantially hindered by fragmented care and poor communication across professionals and settings. While team meetings within settings improved care, communication across settings—such as between primary, secondary, and long-term care—was often insufficient:

“it’s not common to contact the physicians in Serbia, so I couldn’t contact her physician and tell my concerns. So, I advised the patient to go to her physician and to ask about it, but I was not sure if anything happened or changed.” (Multi-country FG4, Pharmacists).

This lack of structured communication and care pathways led to confusion over treatment priorities and clinical responsibility of different professionals about medication review or expertise for decision-making. It generated additional requirements for multiple referrals and consultations.

“because many specialties are involved for a patient, we find it difficult at times to agree with each other on the treatment to be administered.” (Greek FG2, Rehabilitation centre and hospital professionals).

System-level strategies to mitigate fragmentation and improve coordination of care included greater access to allied HCPs, such as psychologists, occupational therapists, and dietitians, and having a clear care team leader were suggested to tackle this. At an institutional level, standardised care protocols, guidelines, documents, referral criteria, and care pathways were recommended, using shared language to facilitate communication.

“Given the absence of formal training, establishing a common language among staff should be the fundamental starting point. Without it, the ability to mobilise an institution towards a unified approach is severely compromised.” (Romanian FG2, Long term care professionals).

### 2c. Structural and contextual challenges: the emerging nature of geriatric care

Multi-country FG geriatricians felt there was systemic underrecognition of GM within the broader medical community, contributing to interprofessional tensions and undermining patient outcomes.

“They [colleagues] are not even aware that geriatric medicine can improve the quality of life of an older person. They are not aware of the fact that we can treat illnesses in the geriatric approach.” (Multi-country FG2, Geriatrics professionals).

In many countries, GM was not recognised as a speciality, contributing to variability in service provision, workforce development, and professional legitimacy.

“In Albania, there is still not a subspecialization in geriatric medicine. At least in North Macedonia, they have developed it this year.” (Multi-country FG1, GPs and internists).

Even in those countries where geriatrics was a speciality, such as Poland, a lack of geriatricians or difficulties accessing them was reported. Participants considered that geriatrics as a profession was a work in progress, needing wider promotion and structural and policy changes such as formal recognition as a medical (sub)speciality, creation of specific wards or academic posts, providing essential services for older people including home and primary care, integration into medical curricula, and raising awareness with professional colleagues. Participants linked the development of geriatric care to broader demographic trends and future health system sustainability.

“We’ve witnessed the shifts in our age demographics and understand the implications. Therefore, failing to act now will have consequences for our children in the years to come.” (Romanian FG2, Long term care professionals).

### 3a. Uncertainties and unmet training needs: lack of an evidence base and context-adapted guidelines

The limited guidance and research available for geriatric care meant that clinicians relied mainly on clinical judgement and experience for complex situations and therapeutic dilemmas. This contributed to variability in care practices, reduced confidence among less experienced staff, and challenges in standardising care.

“There are staff members who don’t know what they’re supposed to do.” (North Macedonian FG2, Specialised geriatric and palliative medicine hospital and nursing home professionals).

As mentioned by the Polish participants, guidelines specific to this population’s complex needs are often lacking or outdated. Participants felt this was partly due to a lack of evidence base to inform guidance and training:

“we do not have clear guidelines, therefore, for these age groups and we are in therapeutic dilemmas.” (Greek FG1 Primary, home care and ambulatory care professionals).

“We love clinical guidelines, but we do not have randomised clinical trials in elderly [sic] people to decide what is the best medicine for this patient.” (Multi-country FG1, GPs and internists).

Beyond medical management, guidance and standardised care protocols on social issues were lacking in some countries: for example, abuse of older people in Greece, or home-based functional support in North Macedonia.

“the manual is literally the algorithm of how I will process the patient in the system. But that patient returns home alone, cannot cook, lives alone, so we enter the social component of the patient.” (North Macedonian FG1 Geriatric specialist care hospital and primary care professionals).

### 3b. Uncertainties and unmet training needs: gaps in existing training

Participants consistently rated their geriatric knowledge and skills as ‘insufficient’ or ‘basic,’ with no consistent pattern across setting, country, or professional. They primarily relied on clinical experience and peer collaboration rather than formal training. As a result, learning in geriatrics was often informal, uneven, and highly dependent on individual initiative rather than structured educational pathways.

“My knowledge [on geriatrics] is only derived from lived experience, not scientific training, it’s incredible!” (Greek FG2, Rehabilitation centre and hospital professionals).

Undergraduate geriatric education was identified as a particularly weak point in geriatric training, lacking sufficient depth and practical experience, especially by participants from countries where relevant modules are optional rather than mandatory, such as Greece and Portugal:

“And I asked myself: Is there no geriatrics course in basic education of doctors? Ehm, no. It is optional…” (Greek FG1 Primary, home care and ambulatory care professionals).

Mandatory geriatrics modules were viewed as a positive next step by professionals from multiple countries. Participants who had received undergraduate geriatric modules considered them beneficial for their clinical practice, but still noted room for improvement.

Post-qualification and continuing professional development opportunities in geriatrics were described as fragmented and inconsistent across countries and professions. While some speciality programmes had emerged in Portugal, Poland, and Greece, many courses were criticised for theoretical emphasis over practical skills. Some training was available to specific HCPs (e.g. geriatric-specific skills for physiotherapists and a nursing speciality in Greece), but other professions lacked clear clinical training (e.g. no geriatric training in Portuguese medical residency programmes or for Polish paramedics). International training experiences were described as particularly valuable for developing practical skills and exposure to alternative care models.

“this year I also had the pleasure of being on such an internship in Japan concerning long-term care for older adults, in 24-h care centers. I could see how it works there.” (Polish FG1, outpatient, primary care and hospital professionals).

The lack of institutional and financial support for geriatric training meant individuals often had to self-fund limited, expensive, or low-quality courses, sometimes requiring long-distance travel to access better options.

“I would like to take the nursing specialty in gerontology but…I can’t because I’m a single parent and I can’t leave my job and go, let’s say, to Patras or…(pause) come to Athens.” (Greek FG1 Primary, home care and ambulatory care professionals).

Furthermore, participants in North Macedonian, Portuguese, Greek and multi-country nursing FGs reported that training was not always formally recognised, did not reliably translate into salary progression or career advancement, and, in one Polish focus group, was associated with increased clinical responsibility for older patients. Some gained knowledge mainly through professional association activities, but this could be problematic if an association did not exist (e.g. in Albania). Taken together, these training gaps contributed to staff shortages and perpetuated HCP GM misconceptions—e.g. viewing geriatric deficits as untreatable or holistic care as unnecessary.

### 3c. Uncertainties and unmet training needs: clinical practice uncertainties

Besides the lack of an evidence base for clinical guidelines and protocol, geriatrics was viewed as a field with inherent scientific uncertainties. Key uncertainties across groups included distinguishing ageing from pathology, knowing when further testing or treatment may do more harm than good, identifying red flags for acute events, and managing medications safely. These uncertainties were particularly problematic in emergency situations where medical history and time for assessment was limited, or in recurrent cases. Important medication uncertainties, particularly in the context of polypharmacy, included de-prescribing, drug interactions, and whether age should be accounted for in medication choice/dose.

“We know when we see a lot of medications, we are alert already. But the thing is, what do we actually do with that and the interactions? And when do we stop something and when do we not?” (Multi-country FG1, GPs and internists).

Uncertainty also extended to professional roles and referral pathways, particularly in relation to complex social care needs. Some professionals in Greek, Polish, and Portuguese and multi-country GPs FGs expressed uncertainty about their own scope of practice for older people as well as where to refer for help with the management of complex social issues.

“we, as healthcare collaborators, also need education on where to refer for the social components.” (North Macedonian FG1, Geriatric specialist care hospital and primary care professionals).

In addition to commonly reported uncertainties, further areas that present special challenges due to management uncertainties included nutrition, cognitive assessment, frailty, delirium, dementia care, fall prevention, multiple chronic disease management, mental health support, pain management, swallowing, speech and occupational therapy, adapting physical activity for older people, ethical dilemmas, and end of life care for older people.

### 3d. Uncertainties and unmet training needs: recommendations for a framework to promote geriatric education

Participants articulated a set of interrelated recommendations to strengthen geriatric education across the training continuum. There was strong support across FGs for geriatrics to be a mandatory part of undergraduate HCP training, including theoretical learning (modules) and practical clinical experience (rotations, placements); this would ensure a minimum of core competencies as a requirement for professional licence. Post-qualification training suggestions included formal specialisation across disciplines, and short GM courses for HCPs of various specialities, which could be broadly targeted, flexible, and accessible, e.g. on-site seminars and conferences. Mandatory or strongly incentivised training, supported by healthcare institutions, employers, and professional geriatrics associations where present, was viewed as necessary to reduce variability in care quality and avoid reliance on individual motivation. 

“Training should be a bit more organized and at an administrative level in our workplaces. That is, to be encouraged and enforced.” (Greek FG2, Rehabilitation centre and hospital professionals).

There was consensus that training courses should include case-based discussions, practical demonstrations, simulations, and role playing, with the option for refresher courses.

“Simulations are very supportive, because if we experience for ourselves what it looks like, we also change our approach and perspective of this older person.” (Polish FG1, outpatient, primary care and hospital professionals).

Interdisciplinary collaboration was identified as a core principle underpinning effective geriatric education, with some FGs favouring interdisciplinary and international approaches to build shared care protocols and strengthen multidisciplinary working.

“We need a multidisciplinary team in order to work together, in order to tailor all these needs.” (Multi-country FG1, GPs and internists)

Certain skills were recognised by most participants as transversal and critical for inclusion in further training for all interdisciplinary healthcare professionals, including good communication with older patients and their families, and mastering of standardised, practical assessment tools to enable a basic geriatric assessment in busy daily clinical practice.

“If we could all, from our own fields, perform the proper geriatric assessment.” (Greek FG2, Rehabilitation and hospital professionals)

Other practical skills discussed by North Macedonian nursing and care professionals included wound care, first aid, safe patient transfer, emergency response, and bedside care.

Ongoing support and mentorship were mentioned in Greek, Polish, North Macedonian and multi-country GP and internist FGs, in the form of regular supervision or team involvement from geriatricians, peer support for case-based discussions, or through working groups in scientific societies. However, a key concern across multiple countries regarding future training was the lack of a critical mass of skilled and qualified professionals available to deliver high-quality courses. This tension between recognised training needs and limited educator capacity was viewed as a critical challenge for implementation.

“Of course, I’ve been wondering all this time about all this training, who’s going to undertake it. I mean, personally, I only know one geriatrician.” (Greek FG2, Rehabilitation centre and hospital professionals).

## Discussion

This multi-country FG study, involving 125 diverse HCPs, provides compelling evidence of a persistent gap between the clinical demands of effective geriatric care and the training currently provided. Taken together, the findings highlight three interrelated domains: clinical complexity in geriatric care, structural barriers within health systems, and substantial gaps in geriatric education and training.

Across countries, HCPs described multiple challenges in managing older adults, often attributing these to insufficient prior training and unmet educational needs in GM. Geriatric care was described as significantly compromised by systemic resource deficiencies, fragmented care pathways, and the evolving nature of GM as a medical speciality. Crucially, the key educational uncertainties that should be addressed in future training include distinguishing physiological ageing from pathology, appropriate assessment tool utilisation, recognising limits of intervention, identifying critical red flags, and comprehensive medication management. Participants’ accounts suggest that these needs could be addressed through mandatory theoretical and practical geriatrics content at undergraduate level. For non-specialists, basic and advanced competency courses were recommended, ideally using interdisciplinary, practical, case-based approaches. While the direction of findings was consistent across groups, the relative importance of specific training gaps varied by professional role and national context.

This qualitative research provided insightful details into the geriatrics educational needs in non-GM specialist HCPs, which is one of the main objectives of the PROGRAMMING project. The holistic and multidisciplinary nature of GM [3], with a strong emphasis on communication with patients and caregivers, was intuitively identified from participants’ practical experience working with older patients. The emotionally demanding but rewarding nature of geriatrics, with its emphasis on relationships and long-term care, could be reframed as a positive asset to increase HCP engagement with training.

Our study identified several structural barriers to effective geriatric care and training including lack of resources, fragmented care, and inconsistent development of GM across Europe. Fragmented care can be overcome by paying attention to the team structure, social processes, formal processes, and team attitudes, along with good information systems, governance, and organisational culture [23]. Although GM is recognised as a speciality in 23 European countries [6], there remains considerable heterogeneity in official recognition and integration within healthcare systems [24]. This variability affects awareness of geriatric principles and potentially the quality of care of older people [25]. Whilst geriatric nursing roles are making good progress in Italy and other countries, there is a notable lack of formal recognition in countries such as Russia [26]. These findings underscore the need for national and institutional reform with clear standards, strategic leadership, and engagement of key stakeholders to drive sustainable change [19]. Engaging with the wide range of relevant stakeholders in European geriatric medicine may help to engender this change [27].

The educational GM gaps identified in the present study concur with previous similar studies. The prevalence, amount, and mandatory nature of geriatrics content in global undergraduate geriatric medical curricula varies substantially [16], as well as within countries [28], likely reflecting the lack of consensus regarding optimal delivery of undergraduate geriatric content [29]. Existing undergraduate medical curricula currently lack sufficient depth on age-related pathophysiology, multimorbidity, and older adults’ social determinants of health [10], whilst gerontological nurse specialists expressed a need for more in-depth training regarding core professional knowledge and clinical skills [11]. HCPs across a range of disciplines expressed interest in further training in our study, reinforcing this gap. European societies have now defined a common core GM speciality curriculum with a list of minimum training requirements and topic areas [6, 30], and PROGRAMMING aims at identifying the most pertinent content of educational and training activities on GM, targeting non-geriatrician HCPs, with a special focus on countries with emerging GM [19]. However, leadership also needs to be a particular focus within GM [31] to overcome the training and mentorship gaps for non-geriatric HCPs. There is also a need to identify interprofessional competencies relevant to all HCPs providing care for older people, as well as profession- or setting-specific competencies. Studies on these topics are currently ongoing as part of the PROGRAMMING COST Action.

Our results indicated that an effective training framework to upskill non-GM HCPs in care of older people needs to involve both theoretical and practical learning, with a strong clinical focus and basic and advanced levels. Previous studies have also indicated a need to integrate comprehensive geriatric content throughout the educational continuum, from undergraduate exposures to specialised postgraduate training [10–15]. For post-qualification training, our results demonstrate the value of an interdisciplinary educational approach that would reflect the multidisciplinary care central to GM and may promote teamwork and reduce fragmentation [32]. However, the best way to deliver this in geriatrics short courses needs further exploration. Although face-to-face approaches were advocated within our FGs, use of online approaches may mitigate the concerns raised regarding distance, timing, quality assurance, expertise availability, and accessibility of courses. Case-based GM e-learning courses have previously been delivered successfully [10]. The most suitable modes of delivery remain an area for future exploration and evaluation. Given the structural barriers, future training initiatives should also leverage innovative methods such as online communities of practice and virtual simulation technologies to increase accessibility and engagement, drawing inspiration from successful methods from countries where GM is well established.

This study has elicited HCPs’ views across various European settings on contemporary geriatric care education, highlighting not only significant educational and clinical care gaps, but also systemic and ethical tensions punctuating everyday practice. We drew on HCPs’ lived experiences and used a consistent approach to data collection across multiple countries and settings. Group dynamics appeared to have a limited effect upon data collection, with reflexivity enhanced through keeping detailed notes on procedures. Most single-country FG facilitators reported participants having an equal chance to speak and few hierarchical issues. Shared professional identities within most single-profession multi-country FGs established rapport and trust (although may have assumed shared knowledge), but the different backgrounds in the GP-internist multi-country FG made uncovering shared needs more challenging. We could not reflect on differences in online and face-to-face dynamics as facilitators were different.

The FG methodology and purposive sampling used limit the generalisability of findings to all healthcare professionals across diverse European contexts. Although it is likely that those taking part in the focus groups were more interested in GM and geriatrics training, they identified a comprehensive range of needs that were relatively consistent across the different groups. The study is likely to be somewhat transferable to other settings where geriatrics is emerging, but different challenges may arise within the unique landscape of other care structures, educational programmes, and regulatory contexts. Participant recruitment was diverse but not exhaustive, and future research could benefit from even broader geographical and professional representation to capture additional nuances in educational needs. Finally, the non-eligibility of professional translations for Action funding gave rise to challenges in cross-language analysis, potentially leading to some loss of nuance.

## Conclusion

Despite well-documented demographic ageing across Europe, there remains a clear educational gap for healthcare professionals caring for older people. The management of the healthcare needs of the older population is complex, and it requires not only clinical guidelines, standardised pathways of care, and protocols but also professionals with specialised geriatric competencies, interdisciplinary working skills, and strong communication abilities. This multi-country focus group study highlighted a clear need for GM training across HCPs, settings and countries. To meet these challenges of uncertainty and complexity, there is a need for innovative, accessible, and practice-orientated training models, covering key areas such as medication management, appropriate prescription, geriatric assessment, physiological mechanisms of ageing, and recognising red flags for critical health events. Training should be accessible and provide practical skills, case-based discussions, and didactic content. To ensure quality, consistency, and availability of geriatrics training, these educational innovations must be supported by structural reforms at national and institutional levels.

## Supplementary Information

Below is the link to the electronic supplementary material.

Supplementary file1 (DOCX 19 KB)

Supplementary file2 (DOCX 26 KB)

## Data Availability

Anonymised focus group transcripts can be made available to those joining or who are members of the Action for any further analyses. Transcripts and permissions to use should be sought in writing from the data owners.
