# Supplementary material for: Assessment of European health professionals’ educational needs in basic principles of geriatric medicine: a focus group qualitative analysis from the PROGRAMMING COST Action 21122
Source: Eur Geriatr Med. 2026 Mar 11;17(3):1269–80. doi: 10.1007/s41999-026-01430-0 (PMC13309381; doi:10.1007/s41999-026-01430-0)
Supplement: Supplementary file 1 — Supplementary file1 (DOCX 19 KB) [file 41999_2026_1430_MOESM1_ESM.docx]

**Supplementary File 1. Detailed summary of focus group composition**

| **Country** | **Focus group (N)** | **Facilitators** | **Gender (F/M)** , **mean age** | **Working settings of participants** | **Profession of participants** |
| --- | --- | --- | --- | --- | --- |
| **Greece** | FG1 (N=10) | M Tampaki (physician)  G Tsamasiotis (physiotherapist) | 9/1  41.1 yrs* | Primary, homecare, ambulatory | Nurses, physical therapists, occupational therapist, health visitor, cardiologist |
|  | FG2 (N=8) | E Mougakou (physician)  E Moumtzi-Nakka (physician) | 6/2  40.0 yrs** | Rehabilitation centre, hospital | Physicians, oncologist, general practitioner (GP), physical therapist, nurse, speech therapist, occupational therapist |
| **Romania** | FG1 (N=8) | AG Prada (geriatrician in training)  C Raluca Nuta (geriatrician) | 7/1  43.1 yrs | Ambulatory care | Other specialties (GP, oncology, endocrinology, cardiology, dermatology) |
|  | FG2 (N=8) | AM Herghelegiu (geriatrician)  OL Bajenaru (geriatrician) | 5/3  45.4 yrs | Long term care | Physiotherapists, nurses, clinical pharmacist, manager, psychologist, GP |
| **North Macedonia** | FG1 (N=12) | P Milosavljevikj (neurologist)  S Arsov (MD, professor and epidemiologist) | 10/2  47.1 yrs | Specialised hospital for geriatric and palliative medicine / LTC, primary care (private GP offices) | Internal medicine, specialist psychiatrist, specialist neurologist, GP, social worker, psychologist |
|  | FG2 (N=12) | V Popov (nurse)  M Terzieva (nurse) | 11/1  46.0 yrs | Specialised hospital for geriatric and palliative medicine, nursing home | Nurses, physiotherapist, caregivers |
| **Portugal** | FG1 (N=10) | A Viegas (family physician with geriatric competence)  S Ganhão-Arranhado (clinical nutritionist, PhD in Gerontology and Geriatrics) | 9/1  43.0 yrs | Primary care | MD, physiotherapist, clinical nutritionist, psychologist, speech therapist, occupational therapist |
|  | FG2 (N=13) | A Farinha (nephrologist with geriatric competence)  J Fonseca (internist with geriatric competence) | 10/3  40.9 yrs | Hospital | Clinical nutritionists, MD, oncologist, nurses, physiotherapist, operational assistant, social assistant |
| **Poland** | FG1 (N=10) | H Lesz-Przybył (geriatrician)  A Kasiukiewicz (internist, geriatrician) | 8/2  44.4 yrs | Outpatient clinics, GP practices, hospitals | Physycians – specialists in geriatric medicine (GM), internal medicine, family medicine; GM trainee; nurses; physiotherapist; paramedic |
|  | FG2 (N=11) | R Kupis (public health specialist, trainee in internal medicine)  M Małek (geriatrician, trainee in internal medicine) | 8/3  30.7 yrs | Hospitals, outpatients clinics, emergency department | Residents of various non-GM medical specialties |
| **Multi-country** | FG1 (N=6) | S Savas (internist with geriatric competence)  ND Yilmaz (medical educator) | 4/2  41.2 yrs | University hospital, primary care, outpatient geriatrics clinic, haemodialysis | GPs, internists |
|  | FG2 (N=6) | S Cotobal Rodeles (geriatrician)  A Christakou (physiotherapist) | 5/1  40.5 yrs | Acute care hospital for older patients, chronic disease hospital, medical hospital for older people | Geriatricians |
|  | FG3 (N=6) | T Yellon (geriatric nurse practitioner)  ND Yilmaz (medical educator) | 5 / 1  40.1yrs | Clinical nursing, nursing academy, nursing faculty | Nurses |
|  | FG4 (N=5) | M Gugu (pharmacist)  M Ortner Hadziabdic (pharmacist) | 5/0  39.2 yrs | Clinical – hospital and community, academia | Pharmacists |

*age data collected as categorical data, 20-40 (5), 40-60 (4) 61+ (1). The midpoint was used to calculate an approximate mean age.

** age data collected as categorical data, 20-40 (4), 40-60 (4), 61+ (0). The midpoint was used to calculate an approximate mean age.
