# Supplementary material for: Assessment of European health professionals’ educational needs in basic principles of geriatric medicine: a focus group qualitative analysis from the PROGRAMMING COST Action 21122
Source: Eur Geriatr Med. 2026 Mar 11;17(3):1269–80. doi: 10.1007/s41999-026-01430-0 (PMC13309381; doi:10.1007/s41999-026-01430-0)
Supplement: Supplementary file 2 — Supplementary file2 (DOCX 26 KB) [file 41999_2026_1430_MOESM2_ESM.docx]

# Supplementary file 2: Coding frameworks

**Cross-country descriptive coding framework**

1. **Current experiences of providing geriatric care**
   1. Description of geriatric care experience
   2. Importance of geriatric care
   3. Goals of geriatric care
      1. Improve quality of life
      2. Holistic and patient centred approach
      3. Prevention
      4. Restoring and promoting health and independence
      5. Restoring sense of purpose
   4. Common issues arising in geriatric care
      1. Clinical conditions
         1. Frailty
         2. Specific conditions
         3. Dependency
         4. Multimorbidity and complexity
         5. Medication management
      2. Psychosocial problems
         1. Depression, despair and lack of motivation
         2. Loneliness
         3. Difficulty adapting to new things
         4. Positive mindset
   5. Management challenges
      1. Case complexity
      2. Time-consuming
      3. Different perspectives on treatment
      4. Medication decisions
   6. Multidisciplinary approach
      1. Importance of different specialities
      2. Importance of teamwork and communication
   7. Structural challenges
      1. Staffing levels
      2. Healthcare system pressures (lack of time, many older people)
      3. Remoteness
      4. Lack of public long term care
      5. Lack of guidelines and policy
   8. Communication with older patients
   9. Interactions with family and caregivers
   10. Emotions in geriatric care
   11. Lack of priority placed on geriatric care
   12. Misconceptions about geriatric care
2. **Knowledge gaps**
   1. Professional uncertainties
      1. Goals and priorities for care
      2. Providing specialised care
      3. Assessment tools
      4. Disease vs physiological ageing
      5. Medication management, ADRs and interactions
      6. Technology
      7. Referrals
      8. Unsure of other healthcare professionals’ skills
   2. Related skills
      1. Addressing wider issues (e.g. depression, self-care, safety)
      2. Communicating with older people and family
   3. Self-rated knowledge and skills level
3. **Training**
   1. Existing training – benefits and issues
      1. Undergraduate training
      2. Postgraduate and CPD courses
      3. Time and resources to attend
      4. Lack of training
   2. Needs
      1. Assessment and evaluation
      2. Biological vs pathological ageing
      3. Drugs and older people (interactions, polypharmacy, complementary therapies)
      4. Communication skills
      5. Managing multiple chronic conditions
      6. Nutrition and diet
      7. Cognition and dementia
      8. Frailty
      9. Mental health
      10. Swallowing
      11. Rehabilitation
      12. Speech therapy
      13. Occupational therapy
      14. Social issues
      15. Adherence barriers for older people
      16. Ethics
      17. Atypical presentations
      18. Palliative care
      19. Functional needs
   3. What training should be like
      1. Undergraduate level education
      2. Postgraduate education
      3. Continuing professional development programmes
      4. Practical training
      5. Interdisciplinary training
      6. International training
      7. Emphasise benefits of geriatric care
   4. Concerns about future training programs
      1. Basic and advanced competencies needed
      2. Individual responsibility for training
      3. Lack of geriatricians for training
      4. Low interest
      5. Challenging discipline with lack of prestige
      6. Need for evidence base
4. **Improving care of older people**
   1. Linking with other organisations
      1. Religious groups
      2. Local social groups
      3. Volunteers
      4. Universities and schools
   2. Standardised guidance
   3. Further multi-professional support and collaboration
   4. Strengthening geriatrics as a specialty
   5. Technology and information sharing
   6. Structural changes (e.g. better referral systems)
   7. Improving communication with patients, relatives and formal caregivers
   8. More focus on medication

**Cross-country codes and themes: Analytical framework**

1. **Current experiences of providing geriatric care**
   1. **Balancing clinical complexity with person-centred care**
      1. Description of geriatric care experience
      2. Importance of geriatric care
      3. Goals of geriatric care
         1. Improve quality of life
         2. Holistic and patient centred approach
         3. Prevention
         4. Restoring and promoting health and independence
         5. Restoring sense of purpose
      4. Common issues arising in geriatric care
         1. Clinical conditions
            1. Frailty
            2. Specific conditions
            3. Dependency
            4. Multimorbidity and complexity
            5. Medication management
         2. Psychosocial problems
            1. Depression, despair and lack of motivation
            2. Loneliness
            3. Difficulty adapting to new things
            4. Positive mindset
      5. Management challenges
         1. Case complexity
         2. Time-consuming
         3. Different perspectives on treatment
         4. Medication decisions
      6. Multidisciplinary approach
         1. Importance of different specialities
   2. **The importance of communication with patients and their caregivers**
      1. Communication with older patients
      2. Interactions with family and caregivers
      3. Emotions in geriatric care
      4. Structural challenges
         1. Lack of public long term care
      5. Improving communication with patients, relatives and formal caregivers
      6. Linking with other organisations
         1. Religious groups
         2. Local social groups
         3. Volunteers
         4. Universities and schools
2. **Structural and contextual challenges**
   1. **Staffing and resource problems**
      1. Structural challenges
         1. Staffing levels
         2. Healthcare system pressures (lack of time, many older people)
      2. Remoteness
   2. **Fragmented healthcare systems**
      1. Multidisciplinary approach
         1. Importance of different specialities
         2. Importance of teamwork and communication
      2. Further multi-professional support and collaboration
      3. Structural changes (e.g. better referral systems)
      4. Technology and information sharing
   3. **The emerging nature of geriatric care**
      1. Lack of priority placed on geriatric care
      2. Misconceptions about geriatric care
      3. Professional uncertainties
         1. Unsure of other healthcare professionals’ skills
      4. Concerns about future training programs
         1. Low interest
         2. Challenging discipline with lack of prestige
      5. Strengthening geriatrics as a specialty
3. **Uncertainties and unmet training needs**
   1. **Gaps in existing training**
      1. Self-rated knowledge and skills level
      2. Existing training – benefits and issues
         1. Undergraduate training
         2. Postgraduate and CPD courses
         3. Time and resources to attend
         4. Lack of training
   2. **Uncertainties to address in future training courses**
      1. Professional uncertainties
         1. Goals and priorities for care
         2. Providing specialised care
         3. Assessment tools
         4. Disease vs physiological ageing
         5. Medication management, ADRs and interactions
         6. Technology
         7. Referrals
      2. Related skills
         1. Addressing wider issues (e.g. depression, self-care, safety)
         2. Communicating with older people and family
      3. More focus on medication
   3. **Lack of an evidence base and guidelines**
      1. Structural challenges
         1. Lack of guidelines and policy
      2. Concerns about future training programs
         1. Need for evidence base
      3. Standardised guidance
   4. **Recommendations for a framework to promote geriatric education**
      1. Needs
         1. Assessment and evaluation
         2. Biological vs pathological ageing
         3. Drugs and older people (interactions, polypharmacy, complementary therapies)
         4. Communication skills
         5. Managing multiple chronic conditions
         6. Nutrition and diet
         7. Cognition and dementia
         8. Frailty
         9. Mental health
         10. Swallowing
         11. Rehabilitation
         12. Speech therapy
         13. Occupational therapy
         14. Social issues
         15. Adherence barriers for older people
         16. Ethics
         17. Atypical presentations
         18. Palliative care
         19. Functional needs
      2. What training should be like
         1. Undergraduate level education
         2. Postgraduate education
         3. Continuing professional development programmes
         4. Practical training
         5. Interdisciplinary training
         6. International training
         7. Emphasise benefits of geriatric care
      3. Concerns about future training programs
         1. Basic and advanced competencies needed
         2. Individual responsibility for training
         3. Lack of geriatricians for training
